# Whole genome sequencing identifies a novel homozygous exon deletion in the *NT5C2* gene in a family with intellectual disability and spastic paraplegia

**DOI:** 10.1038/s41525-017-0022-7

**Published:** 2017-06-01

**Authors:** Hossein Darvish, Luis J. Azcona, Abbas Tafakhori, Mona Ahmadi, Azadeh Ahmadifard, Coro Paisán-Ruiz

**Affiliations:** 1 Department of Medical Genetics, School of Medicine, Shahid Beheshti University of Medical Sciences, Tehran, Iran; 2 Department of Neurosciences, Icahn School of Medicine at Mount Sinai, One Gustave L. Levy Place, New York, NY 10029 USA; 3 Department of Neurology, School of Medicine, Imam Khomeini Hospital and Iranian Center of Neurological Research, Tehran University of Medical Sciences, Tehran, Iran; 4 Departments of Neurology, Psychiatry, and Genetics and Genomic sciences, Icahn School of Medicine at Mount Sinai, One Gustave L. Levy Place, New York, NY 10029 USA; 5 Mindich Child Health and Development Institute, Icahn School of Medicine at Mount Sinai, One Gustave L. Levy Place, New York, NY 10029 USA; 6 Friedman Brain Institute, Icahn School of Medicine at Mount Sinai, One Gustave L. Levy Place, New York, NY 10029 USA

## Abstract

Hereditary spastic paraplegias are a rare group of clinically and genetically heterogeneous neurodegenerative diseases, with upper motor neuron degeneration and progressive lower limb spasticity as their main phenotypic features. Despite that 76 distinct loci have been reported and some casual genes identified, most of the underlying causes still remain unidentified. Moreover, a wide range of clinical manifestations is present in most hereditary spastic paraplegias subtypes, adding further complexity to their differential clinical diagnoses. Here, we describe the first exon rearrangement reported in the *SPG45/SPG65* (*NT5C2*) loci in a family featuring a complex hereditary spastic paraplegias phenotype. This study expands both the phenotypic and mutational spectra of the *NT5C2*-associated disease.

## Introduction

Hereditary spastic paraplegias (HSPs) are a rare group of genetically heterogeneous neurodegenerative disorders, characterized by a progressive lower limb spasticity and weakness that results from a loss of corticospinal motor tract function.^1^ Over 70 different HSP loci have already been reported, with several patterns of inheritance, including autosomal recessive (AR), autosomal dominant, and X-linked, being identified. Based on their accompanying clinical symptoms, HSPs are classified in two distinct categories: pure and complex. In complex HSP, intellectual disability (ID), epilepsy, ataxia, optic atrophy, deafness, peripheral neuropathy, and skin abnormalities might be observed. Most complicated forms are usually inherited in a recessive fashion.^2^

In this study, we report the identification of a novel mutation in the *NT5C2* gene (MIM# 613162), also known as *SPG45/SPG65*, in a family presenting with a complex form of AR-HSP. The *NT5C2* locus was first mapped to chromosome 10q24.3-q25.1 in a large AR family with five members featuring complex HSP. The disease was characterized by onset at birth, spastic gait, ID, optical atrophy, and visual defects.^3^ In 2014, the causal gene was cloned by the identification of pathogenic *NT5C2* mutations in five different families.^4^ An additional family carrying a novel homozygous splice-site mutation has recently been reported.^5^

## Results

### Family report

**Fig. 1:**
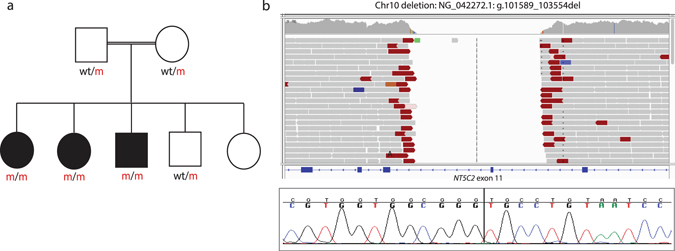
**a** Pedigree structure of a family presenting with complex HSP due to a pathogenic *NT5C2* mutation. Homozygous mutation carriers are represented as m/m and heterozygous carriers as wt/m. *Dark squares* (males) and *circles* (females) indicate HSP. **b** WGS reads of an affected sibling showing the 1.9 kb chromosome 10 deletion (c.771 + 573_814-298del) identified in all affected members are shown at the *top*, while Sanger chromatogram sequences corresponding to the deletion are shown at the *bottom*

A consanguineous Iranian family with three affected siblings was referred to us (Fig. 1a). All three patients presented to our clinic with pronounced gait disturbances. Their mother had normal pregnancy with normal vaginal delivery, and all patients were normal at birth with no evidence of abnormality. The patients had delayed developmental milestones and ID became prominent with age. Walking began in about age 3 but gradually became spastic and problematic. There was no history of epileptic attacks, skeletal or facial deformity, failure to thrive, and no self-mutilation or aggression. There was no increased rate of infectious diseases or any skin abnormality. During examination, the patients showed ID but could perform visual contact and, to some extent, verbal contact. There was marked dysarthria and hypophonic speech, but other cranial exams were within normal limits. Spasticity of the limbs was observed during motor examination, with mild symmetric spastic paraparesis and increased deep tendon reflexes. Babinski sign was present and there was preserved abdominal-cutaneous reflex, but neither tremor nor dystonic features were observed. Coordination was normal and their gait was spastic. Although patients were not cooperative for sensory testing, no prominent sensory abnormality was detected. A summary of the clinical details can be found in Table 1.

**Table 1:** Clinical details of reported patients with *NT5C2*-associated disease

Age at onset	Disease duration	First symptoms	Spasticity	Intellectual disability	Ocular abnormalities	Deep tendon reflexes	Plantar reflex	Other symptoms	MRI	References
Infancy	22–33 years	Gait abnormalities	+	+	Primary optical atrophy, myopia, congenital nystagmus	Increased	Upward	–	Normal (or not done)	3 ^a^
14 months – 4 years, Infancy	3–33 years	Gait abnormalities	+	+	Primary optical atrophy, glaucoma, squint, congenital cataract	Increased	Upward	Aggressiveness, short stature, underweight	Thin corpus callosum, white matter changes	4
7–10 months	3–9 years	Lower limbs spasticity, developmental delay	+	+	Squint, hypermetropy	Increased	Upward	Equinus foot, brownish discoloration, lordosis	Thin corpus callosum, white matter changes, frontal cerebral atrophy	5
Infancy	17–30 years	Gait abnormalities	+	+	Myopia	Increased	Upward	Marked dysarthria, hypophonic speech	Normal	This report

^a^ The *NT5C2* mutation in this family is not yet reported, but this family was the first reported family to be associated with the SPG45/SPG65 locus and that’s why is included in this table

### Homozygosity mapping (HM)

Due to the AR inheritance pattern and the observed consanguinity in the affected pedigree (Fig. 1a), the HM method was used for disease loci identification. Eight different homozygous segments located on chromosomes 7, 8, 10, and 11 were identified to be shared by all affected individuals and to be present as heterozygous regions in non-affected family members (Supplementary Table 1).

### Whole genome sequencing (WGS)

To reduce the number of disease-associated loci and assist with the disease gene identification, WGS was carried out in two affected siblings. After excluding splice-site, coding non-synonymous, small insertions and deletions, as well as stop gained/loss mutations as the disease-causing mutations, copy-number analysis of the WGS data was carried out. A novel, 1954-bp homozygous deletion at the *NT5C2* locus involving the entire coding exon 11 was identified to be present in both sequenced, affected siblings. This deletion (NG_042272.1: g.101589_103554del), found to be located in a previously identified, disease-associated locus at chromosome 10 (Supplementary Table 1), resulted in c.771 + 573_814-298del nucleotide change and p.(Lys258_Lys271del) amino-acid change (Fig. 1b, Table 2). Subsequently, the examination of the deletion’s flanking regions in the remaining family members through Sanger sequencing revealed that this deletion was present in homozygous state in all affected members, while the three available unaffected cases were found to be heterozygous deletion carriers. This was later verified by the amplification of the *NT5C2* exon 11 in all family members, which certified the absence of the exon 11 in all affected members and its presence in the healthy family members (Fig. 1a), further confirming its segregation with the disease status. The *NT5C2* exon 11 was found to be present in the 50 different *NT5C2* transcripts reported in NCBI website (https://www.ncbi.nlm.nih.gov/gene/22978?report=full_report), further supporting its presence in all reported human transcripts. No additional mutation was identified in the remaining disease-associated loci.

**Table 2:** All *NT5C2* mutations reported in HSP families

Locus (gene)	Nucleotide change	Amino-acid change	Reference
SPG45 (NT5C2)	c.86 G > A	p.Arg29Stop	4
SPG45 (NT5C2)	c.175 + 1 G > A	N/A	4
SPG45 (NT5C2)	c.445 A > T	p.Arg149Stop	4
SPG45 (NT5C2)	c.989 – 1 G > T	N/A	4
SPG45 (NT5C2)	c.1159 + 1 G > T	N/A	5
SPG45 (NT5C2)	c.1225delA	p.Ser409Valfs436Stop	4
SPG45 (NT5C2)	c.771 + 573_814-298del	p.(Lys258_Lys271del)	This report

## Discussion

We describe the identification of a novel *NT5C2* mutation in a family with complex AR-HSP. By combining both linkage and WGS data, we were able to identify a large homozygous 1954-bp *NT5C2* deletion as the causative genetic variation for a complex form of HSP. The clinical phenotype, which resembles previous reported cases, is characterized by an infantile onset, ID, delayed walking, increased deep tendon reflexes, and pronounced gait disturbances. Unlike other reported cases, no skeletal deformities were observed. While marked dysarthria and hypophonic speech, not previously reported in *NT5C2*-associated disease,^3–5^ were present in all three patients (Table 1).

We here report the first *NT5C2* exon rearrangement in a family with complex HSP. Six other *NT5C2* mutations, including slice-site, nonsense, and frameshift mutations, were previously reported in complex HSP (Table 2). We here report the first exon rearrangement described at the *NT5C2* locus. Given the high frequency of exon rearrangements in other complex AR-HSP genes, such as *SPG11*,^6^ it is not surprising to identify these types of mutations in this recently identified HSP gene. However, copy number variations (CNVs) are more likely to be captured through WGS, rather than whole exome sequencing, as WGS captures both coding and non-coding genetic variation, allowing us to map the CNV breakpoints, and leads to improved detection of CNV and de novo variations due to its read coverage uniformity and allele reduced bias.^7, 8^ Therefore, with the growth and availability of WGS technology, we are confident that more CNVs would be mapped and identified at the *NT5C2* and other HSP loci, which will certainly aid in the differential diagnosis of this heterogeneous group of neurodegenerative diseases.

This report expands both the phenotypic and mutational spectra of the *NT5C2*-associated disease.

## Material and methods

### Subjects

A consanguineous family with AR-HSP was clinically examined (Fig. 1a). The local ethics committee at the Shahid Beheshti University of Medical Sciences approved this study, and informed consent according to the Declaration of Helsinki from all participants was obtained. DNA samples from all members were isolated from whole blood using standard procedures.

### Homozygosity mapping

High-throughput single nucleotide polymorphism genotyping was carried out in all available family members (*n* = 6) (Fig. 1a) using the HumanOmniExpress Exome arrays v1.3 and HiScanSQ system (Illumina Inc., San Diego, CA, USA). The GenomeStudio program (GS; Illumina) was used to undertake quality assessments and generate PLINK input reports^9^ for HM, and HM analyses were carried out as previously described.^10, 11^

### Whole genome sequencing

Two affected family members were subjected to WGS analyses. WGS was carried out at the New York Genome Center. Sequencing libraries were constructed with the TruSeq PCR-free library kit (Illumina) following the manufacturer’s recommended protocol. Libraries were sequenced on the Illumina HiSeq X instruments, with 2 × 150 bp paired reads, to a minimum coverage of >30×. Sequencing data was processed as previously described.^11^

### Validation and disease-segregation

The *NT5C2* deletion identified through WGS was validated through direct Sanger sequencing by using primers flanking the deletion breakpoints and primers amplifying the *NT5C2* exon 11. Primer sequences were designed by using a public primer design website (http://ihg.gsf.de/ihg/ExonPrimer.html) (sequences available upon requested). All purified PCR products were sequenced in both forward and reverse directions with Applied Biosystems BigDye Terminator v3.1 sequencing chemistry as per the manufacturer’s instructions, and resolved and analyzed as described elsewhere.^10^

### Data availability

The DNA variation identified in this study is deposited in Open source databases, such as The Leiden Open Variation Database (http://databases.lovd.nl/shared/phenotypes/0000073477) and the ClinVar NCBI database (https://submit.ncbi.nlm.nih.gov/subs/clinvar_wizard/SUB2299204/overview). The raw data that support the findings of this study are now available from the corresponding author upon reasonable request, and will be deposited in dbGAP upon completion of the grant proposal.

### Ethical approval

Ethical approval was obtained from the Institutional Review Board of the Shahid Beheshti University of Medical Sciences and all methods were performed in accordance with relevance guidances and regulations.

## Electronic supplementary material

Supplementary Table

Patient consent form - blank (in Persian)
