# Supplementary material for: Whole genome sequencing identifies a novel homozygous exon deletion in the NT5C2 gene in a family with intellectual disability and spastic paraplegia
Source: NPJ Genom Med. 2017 Jun 1;2:20. doi: 10.1038/s41525-017-0022-7 (PMC5675118; doi:10.1038/s41525-017-0022-7)
Supplement: Supplementary file 1 — Supplementary Table [file 41525_2017_22_MOESM1_ESM.docx]

**Supplementary Table S1: Homozygous chromosomal segments identified to be shared by all affected HSP siblings**

| **Chr** | **SNP1** | **SNP2** | **Position 1 (bp)** | **Position 2 (bp)** | **Size (kb)** |
| --- | --- | --- | --- | --- | --- |
| 7 | rs10228761 | rs1167794 | 73,826,693 | 75,172,270 | 1,345.58 |
| 7 | exm646450 | exm2258040 | 102,079,461 | 103,338,468 | 1,259.01 |
| 8 | rs2458064 | rs309607 | 114,956,591 | 116,118,689 | 1,162.1 |
| 10 | rs7917824 | rs10508822 | 31,901,584 | 36,235,526 | 4,333.94 |
| 10 | rs1926131 | rs10870381 | 37,583,900 | 39,058,671 | 1,474.77 |
| 10 | rs11006408 | rs4933814 | 60,995,585 | 83,755,315 | 22,759.73 |
| **10** | **rs7923326** | **rs11198165** | **97,764,765** | **119632453** | **21,867.688** |
| 11 | rs7110905 | rs1352864 | 24,033,297 | 27,866,783 | 3,833.49 |

The disease-associated locus is represented in bold
