# Supplementary material for: Whole genome sequencing identifies a novel homozygous exon deletion in the NT5C2 gene in a family with intellectual disability and spastic paraplegia
Source: NPJ Genom Med. 2017 Jun 1;2:20. doi: 10.1038/s41525-017-0022-7 (PMC5675118; doi:10.1038/s41525-017-0022-7)
Supplement: Supplementary file 2 — Patient consent form - blank (in Persian) [file 41525_2017_22_MOESM2_ESM.doc]

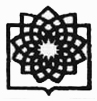


**فرم رضايت نامه آگاهانه براي انجام طرح تحقيقاتي**

**آنالیز هشت ژن ایجاد کننده فرم اتوزومی مغلوب بیماری اتیسم در خانواده های ایرانی**

**آقاي/ خانم محترم**

**ما از شما دعوت مي كنيم تا دريك پروژه پژوهشي شركت نماييد. ما اهميت بالقوه اين پژوهش را باور داريم. با اين حال قبل از آنكه شما تصميم بگيريد در آن شركت كنيد يا خير, ما نياز داريم مطمئن شويم كه شما فهميده­ايد اول اينكه ما براي چه اين پژوهش را انجام مي­دهيم. دوم اينكه اگر موافقت كنيد براي شما /فرد تحت قيموميت شما چه منافع دربرخواهد داشت لطفا" اين متن را به دقت بخوانيد و هر سئوالي كه داريد با اطمينان بپرسيد و اگر تمايل داريد، با خويشاوندان، دوستان يا هر فرد ديگر به بحث و تبادل نظر بپردازيد. ما سعي خواهيم كرد تا بهترين توضيح را ارائه دهيم و هر اطلاعات بيشتري كه شما بخواهيد چه در حال، چه در آينده فراهم آوريم. شما مجبور به اخذ يك تصميم فوري نيستيد. پژوهشگر ارشد/مسئول**

**اينجانب …………… شخصا" يا به نمايندگي از سوي …………… بعنوان ولي/قيم/وكيل قانوني…………رضايت قلبي خود را مبني بر شركت من/موكل/فرد تحت قيموميت من در اين پژوهش بعنوان نمونه/سوژه/بيمار اعلام نموده و موارد ذيل مورد تائيد من مي باشد:**

1. **من مطلع ام كه مجري پروژه بنام دكتر حسین درویش با رتبه علمي استادیار عضو هيات علمي دانشگاه علوم پزشکی شهید بهشتی مي باشد.**
2. **من مطلع ام كه هدف اين تحقيق بررسي علل ژنتيك اوتیسم با یا بدون عقب ماندگي ذهني مي باشد. و مجري روش انجام پژوهش و نحوه انتخاب من/موكل/فرد تحت قيموميت من بعنوان نمونه/سوژه /بيمار را در اين پژوهش به من شرح دادند. ضمنا" به من مهلت داده شد تا ظرف48 ساعت نظر خود را پس از مشورت با هر كس كه مايل هستم ,مبني بر شركت با ميل خود و كاملا" اختياري يا عدم شركت در پژوهش مذكور اعلام نمايم.ضمنا" مجري يادآور شدند كه در صورت اعلام عدم تمايل به همكاري در اين پژوهش رابطه درماني من/موكل/فرد تحت قيموميت من با مركز درماني و پزشك معالج دچار اشكال نخواهد شد .**
3. **مجري منافع و فوائد بالقوه پژوهش مذكور شامل شناسايي علت بيماري در صورت دستيابي به ژن درگير و شناسايي جهش مربوطه در خانواده، انجام مشاوره ژنتيك ، تشخيص و پيشگيري از تولد افراد مبتلاي جديد و مضرات و خطرات احتمالي آن شامل درد و ناراحتي حاصل از نمونه‌گيري را يادآور شده اند.**
4. **اينجانب مي دانم كه اطلاعات مربوط به من/موكل/فرد تحت قيموميت من اعم از اطلاعات شخصي و آنچه مربوط به بيماري يا روش درمان من/موكل/فرد تحت قيموميت من ميشود صرفا" نزد محقق ارشد اين تحقيق قرار دارد و اين محقق به هيچ عنوان اجازه انتشار اطلاعات شخصي من/موكل/فرد تحت قيموميت من را مگر با اجازه كتبي من ندارد و فقط نتايج كلي و گروهي اين تحقيق را مي توانند بصورت مقاله ، گزارش همراه با چاپ اطلاعات كلينيكي و تصاوير خود و خانواده ام در مجله هاي علمي منتشر نمايند. و عنوان کردند که تحقیقات مکرری بر روی نمونه ها انجام خواهد شد.**
5. **به مجري تاكيد كردم علاوه بر بيماري اصلي كه علت مراجعه به اين مركز درماني مي باشد داراي وضعيت هاي خاص يا بيماري هاي مثل سرطان، فاويسم، آسم و ……………… مي باشم.**
6. **مجري آدرس و شماره تماس خود كه اوين- بلوار دانشجو- خيابان كودكيار- گروه ژنتیک پزشکی دانشگاه علوم پزشکی شهید بهشتی – شماره تماس 23872572 مي باشد را در اختيارم گذاشته تا هر وقت كوچكترين مشكلي يا سئوالي در رابطه با شركت من/موكل/فرد تحت قيموميت من در پژوهش مذكور پيش آمد با ايشان درميان بگذارم و راهنمايي بخواهم يا از آخرين اطلاعات در خصوص بيماري/وضعيت خاص خود /موكل/فرد تحت قيموميت من در حين مطالعه مطلع گردم.**
7. **مجري به من/موكل/فرد تحت قيموميت من تاكيد كردند كه تا رسيدن به نتايج نهايي هرگونه آزمايش انجام شده به طور مرتب جهت پيگيري به گروه ژنتیک پزشکی دانشگاه علوم پزشکی شهید بهشتی مراجعه نمايم. ضمنا" مجري اعلام كردند هزينه هاي ناشي از شركت در اين پژوهش با هماهنگي گروه ژنتیک پزشکی دانشگاه علوم پزشکی شهید بهشتی و يا مستقيما"از طريق مجري از محل اعتبار طرح قابل پرداخت خواهد بود. و هزينه اي متوجه من، موكلم، افراد تحت قيموميت من نخواهد بود.**
8. **مجري به من/موكل/فرد تحت قيموميت من تاكيد كردند اگر در حين و بعد از انجام پژوهش هر مشكل اعم از جسمي/روحي/مادي كه ناشي از انجام پژوهش مذكور باشد, براي من/موكل/فرد تحت قيموميت من پيش آمد مي توانم با هماهنگي ايشان به مركز درماني نزديك و مرتبط مراجعه و در صورت لزوم بستري شدن هزينه درمان و غرامت قابل پرداخت به من/ولي/وكيل/قيم/خانواده من يا مركز درماني كه در آن بستري شده است، مي باشد.**
9. **به من/موكل/فرد تحت قيموميت من تفهيم شد اگر شكايتي از مجري يا همكاران يا روند مطالعه دارم مي توانم با مركز قضايي تماس گرفته و بصورت شفاهي يا كتبي شكايت نمايم. هشت مورد متن رضايت نامه و نه نكته مندرج در ذيل مورد تائيد اينجانب مي باشد.**

امضا

**­­**
